# Biochemical but not imaging parameters are predictive of outcome in septic shock: a pilot study

**DOI:** 10.1186/s12947-022-00276-3

**Published:** 2022-03-24

**Authors:** Oriana E. Belli, Jonica Campolo, Paola Vallerio, Francesco Musca, Antonella Moreo, Alessandro Maloberti, Marina Parolini, Luca Bonacchini, Gianpaola Monti, Andrea De Gasperi, Roberto Fumagalli, Cristina Giannattasio

**Affiliations:** 1 De Gasperis Cardio Center, Cardiothoracovascular Department, ASST Great Metropolitan Hospital Niguarda, Milan, Italy; 2 CNR Institute of Clinical Physiology, ASST Great Metropolitan Hospital Niguarda, Milan, Italy; 3 Emergency Department, ASST Great Metropolitan Hospital Niguarda, Milan, Italy; 4 Servizio di Anestesia e Rianimazione I, ASST Great Metropolitan Hospital Niguarda, Milan, Italy; 5 Servizio di Anestesia e Rianimazione II, ASST Great Metropolitan Hospital Niguarda, Milan, Italy; 6 School of Medicine and Surgery, Milano-Bicocca University, Milan, Italy

**Keywords:** Septic shock, Intensive care, Echocardiography, Biomarkers

## Abstract

**Background:**

Septic shock is a severe form of sepsis marked by hypotension with an ominous outcome despite the introduction of modern intensive care. The aim of the present study is to obtain a panel with biomarkers, echocardiographic and vascular parameters to better risk stratify patients and identify those at higher risk of ominous outcome.

**Methods:**

Between May 2013 and April 2016, 35 consecutive patients admitted at the Intensive Care Unit (ICU) of ASST Great Metropolitan Hospital Niguarda with the diagnosis of severe sepsis or septic shock were enrolled. All patients underwent rest echocardiography and several circulating biomarkers of myocardial damage or oxidative stress.

**Results:**

The multivariate Cox’s proportional hazard model showed that the only independent prognostic predictor for 30-day mortality was the angiopoietin-2, (HR 1.017, 95% CI 1.000–1.034; *P* = 0.049). An angiopoietin-2 concentrations ≥ of 33,418 pg/mL was identified as the optimal threshold for the discrimination between survivors and non survivors at the time of admission in ICU, with a sensitivity of 80% and a specificity of 68%.

**Conclusions:**

Septic shock has a poor in-hospital outcome even when the best of care is implemented. Among the biochemical parameters angiopoietin was able to identify patients at risk of death. The lowest the value at admission, the highest the risk of in-hospital death. No echocardiographic nor vascular parameter was able to predict outcome in this setting.

## Introduction

Septic shock is a severe form of sepsis marked by hypotension with an ominous outcome despite the introduction of modern intensive care. The incidence of the condition has been growing over the last few years and, in the US, it reached 1% of all intensive care units (ICU) admittance [1, 2]. The clinical manifestations of sepsis are highly variable depending on several factors such as the site of infection, the overall status of the patient, the germs causing the disease.

Systemic response to sepsis results in a complex chain of events that involve the inflammatory, coagulation, and vascular endothelial systems. Several pro-inflammatory cytokine such as tumor necrosis factor-α and interleukin-6 (IL-6) are important mediators in the pathophysiology of the disease and are responsible for the initial innate immune system response [3]. Cytokines contribute to fever, activate endothelial cells, attract circulating poly-morphonuclear, and enter the circulatory system.

Endothelial dysfunction plays an important role in the pathogenesis of sepsis. In fact, the endothelium plays a key role in the development of macro and microcirculatory disturbances in sepsis [4]. Several factors contribute to diminished oxygen delivery is septic shock. Inflammation can cause dysfunction of the vascular endothelium, accompanied by cell death and loss of barrier integrity favouring a pro-coagulant state. Cardiovascular compromise is manifested primarily as hypotension or an elevated serum lactate level. After adequate volume expansion, hypotension frequently persists, requiring the use of vasopressors, and myocardial dysfunction may occur.

Although several pathophysiologic mechanism involved in sepsis and septic shock have been clarified as well as several biomarkers involved, there is no single clinical or biological parameter able to identify patients at higher risk of death. Previous studies have shown that in severe sepsis and septic shock, left ventricular (LV) diastolic dysfunction and reduced LV volumes are common and predict mortality better than systolic dysfunction [5]. Furthermore, aortic stiffness has recently been indicated as an experimental parameter able to identify early circulatory alterations in sepsis leading to multi-organ failure and increased mortality [6]. The aim of the present pilot study was to assess whether biochemical, echocardiographic and vascular variables could determine a better patients risk stratification, identifying those at higher risk of negative outcome. The study hypothesis is that biomarkers may be better suited to this aim when compared to echo and/or vascular parameters. Systolic and/or diastolic dysfunction is the net effect of the multi-organ failure due to inflammatory biomarkers and not the cause of shock. The interplay between these clinical and biological markers remains elusive.

## Methods

### Patients

Between May 2013 and April 2016 35 consecutive patients admitted at the ICU of ASST Great Metropolitan Hospital Niguarda with the diagnosis of severe sepsis or septic shock were enrolled. Patients were enrolled in the study within 24 h of admission in ICU. Severe sepsis was defined in the presence of all three of the following criteria: (i) evidence of infection or serious clinical suspicion for infection; (ii) at least two signs of the signs of systemic inflammatory response syndrome: (a) temperature > 38 °C or < 36 °C; (b) pulse > 90 b.p.m.; (c) respiratory rate > 20 breaths/min or mechanical ventilation; (d) white blood cells > 12,000 or < 4000 or > 10% bands; and (iii) at least one organ dysfunction [7]. Septic shock was defined as severe sepsis plus hypotension (systolic BP < 90 mmHg) lasting more than 1 h, not responding to fluid therapy (raising central venous pressure to 12 or 15 mmHg in patients with oliguria) and requiring vasopressor therapy [8].

Excluded were patients with greater than mild mitral and/or aortic valve disease, patients with echocardiographic evidence of regional myocardial wall motion abnormality suggesting regional ischaemia or previous infarction and patients with poor quality echocardiographic images and measurements. Informed consent to participate in the study was obtained in all patients or from a relative in case of inability to sign it. The study was approved by the local Ethics Committee.

### 2D echocardiography

At the enrolment, each patient underwent two-dimensional (2D) echocardiography, followed by color flow imaging, pulsed and continuous wave Doppler ultrasound study, and Tissue Doppler Imaging (TDI) for echocardiographic evaluation of myocardial function Two expert operators, working in the high volume ecocardiographic center of Niguarda Hospital, performed the examinations.

Transthoracic echocardiographic studies were performed with commercially available ultrasound machine (Philips CX50 Ultrasound, Andover, Mass, USA and GE Vivid q Healthcare, Wauwatosa, WI, USA) equipped with 2.5–3.5 MHz phased-array sector scan probe and with second harmonic technology. Left atrial, left ventricular (LV) end-diastolic and end-systolic diameters were measured from the 2D echocardiographic images obtained by parasternal long axis view. LV volumes were measured and ejection fraction obtained by two-dimensional and four and two chambers view using modified biplane Simpson’s method, according to the recommendations of the American Society of Echocardiography [9].

Diastolic function was assessed by trans-mitral patterns, TDI analysis on lateral and septal mitral annulus. No other diastolic parameters were analyzed due to poor acoustic window quality or presence of tachycardia. For the same reason, no other functional variables like strain and strain rate have been assessed. Pulsed mitral Doppler measurements were obtained with the transducer in the apical four-chamber view by positioning a 1–2 mm sample volume between the tips of the mitral valve leaflets in diastole, with the Doppler beam aligned perpendicular to the plane of the mitral annulus. We derived early peak filling velocity (E), late peak filling velocity (A), early to late filling ratio (E/A), and mitral E/e’ ratio. Transmitral diastolic patterns was classified in: abnormal relaxation, pseudonormal and restrictive. Severe diastolic dysfunction was defined as the presence of restrictive transmitral pattern and E/e’ average ratio > 14 [10].

Mitral regurgitation has been classified according to the recommendations from the European Association of Echocardiography, and grading in: mild, moderate and severe [11].

### Arterial stiffness

Carotid-femoral pulse wave velocity (cfPWV) and carotid-radial pulse wave velocity (crPWV) were measured at the enrolment by an automatic device (Complior, Artech, France) capable of assessing the rapid upstroke of the foot of arterial pulse wave.

With the patient supine, we measured separately both cfPWV and crPWV. For the assessment of cfPWV, we recorded blood pressure (BP) waveforms from common carotid to femoral artery. The 80% carotid-femoral artery distance was measured by a rigid rule, according to current international guidelines [12], and the cfPWV was calculated as the ratio between this value and the pulse transit time. For crPWV, the BP waveforms were recorded at right side from right common carotid to right radial artery and at left side from left common carotid to left radial artery. The carotid-radial distance was similarly calculated by a rigid rule and the crPWV was calculated as the ratio between this value and the pulse transit time. Pulse transit time was always determined from the average of 10 consecutive cardiac beats to collect data on a complete respiratory cycle, and the mean of the 2 complete cardiac beat was used for analysis. In our laboratory, the intra-session within- and between-operator variability of the cfPWV and crPWV values, expressed as coefficient of variation of the mean, are 3 and 4%, respectively. The within-operator variability between sessions is 4%. Aortic stiffness was defined as a cfPWV measurement > 10 m/s accordingly to current guidelines [12].

### Biological sample collection

Blood and urine samples were collected within 24 h from ICU admission and immediately treated and stored at − 80 °C for the biochemical analyses. The following biomarkers of inflammation, oxidative stress and endothelial activation were analyzed.

Inflammatory markers included plasma interleukin (IL)-6 and others markers involved in the regulation of IL-6 bioactivity, such as soluble IL-6 receptor (sIL-6R) and soluble GP-130 (sGP-130), and urinary neopterin (uNEO) which is an index of immune system activation.

Oxidative stress markers were aminothiols [cysteine (Cys), cysteinylglycine (CysGly), homocysteine (Hcy), glutathione (GSH)], that are indices of oxidant/antioxidant balance, malondialdehyde (MDA) and 3-nitrotyrosine (3-NT), markers of lipid peroxidation and peroxynitrite-mediated nitration, respectively.

Endothelial activation markers were angiopoietin-1 (Ang-1) and angiopoietin-2 (Ang-2) that play an important role in vascular integrity and injury.

### Laboratory measurements

Total plasma (TP) concentrations of Cys, CysGly, Hcy, and GSH were measured according to an high performance liquid chromatographic (HPLC) method validated in our laboratory. Reduced blood (RB) GSH level was instead assessed by mixing whole blood or plasma with 10% of tri-chloroacetic acid (1:1 v/v) before sampling and analysis, as previous described [13].

Plasma MDA was determined by a HPLC commercial kit (ChromSystems, Gräfelfing, Germany) with fluorescence detection (515 nm λ excitation and 553 nm λ emission) while plasma 3-NT, IL-6, sIL-6R, sGP-130, Ang-1 and Ang-2 concentrations by ELISA commercial kits (Hycult Biotech, Uden, The Netherlands, and R&D Systems, Minneapolis, USA).

uNEO levels were instead measured by an isocratic HPLC method previously reported by Caruso [14] and were normalized by urine creatinine concentrations.

A general laboratory biochemical assessment was made in serum [blood cells count, creatinine, estimated gromerular filtration rate, glucose, total/indirect bilirubin, AST, PT, INR, cardiac troponin T (TnT), creatine kinase MB (CK-MB), pro B-type natriuretic peptide (NT-proBNP), pro-calcitonin (PCT), C-reactive protein (CRP)]. All parameters were determined using standard laboratory methods.

### Statistical analysis

Continuous variables are presented as median and interquartile range (I; III) and categorical variables as frequency and percentage. Data were tested for normality of distribution by the Kolmogorov–Smirnov test.

Pearson correlation test or Spearman rank–order correlation test, for non normally distributed variables, were used to correlate baseline specific biochemical variables. Univariate Cox’s proportional hazards regression models was used to identify the strongest predictors of 30-day mortality; only the variables with statistically significant association with mortality on univariate analysis (*P* < 0.10) were included in the multivariate models with the backward selection method. A receiver operating characteristic (ROC) curves were used to assess the optimal (highest combination of sensitivity and specificity) cut-off scores on the variable for discriminating between the outcome. They also permitted calculation of area under the ROC curve (AUC) with their 95% confidence interval (CI).

The Kaplan-Meier method was used for survival analysis, and the log-rank test was used to compare survival curves.

Statistical analyses were performed using SPSS ver. 24.0 software package (IBM SPSS, New York, USA). A *p*-value of less than 0.05 was considered significant. The only end-point analyzed was 30-day.

## Results

The median age of our population was 59 (48; 60) years and they were largely males (60%). The main sources of sepsis were: gastrointestinal, 10 (29%); autoimmune 1 (3%), respiratory, 7 (20%); vascular surgery/limb ischaemia, 1 (3%); genitourinary 5 (14%); orthopaedic/skeletal, 1 (3%) and haematological 10 (29%).

At least one source of infection was identified by positive cultures in 22 (63%) patients.

All patients had a circulatory shock and septic shock persisted despite fluid resuscitation requiring one or more vasoactive medications: norepinephrine, 27 (77%) patients; epinephrine, 22 (63%) patients; dopamine/dobutamine, 2 (6%) patients.

Baseline clinical and anthropometric characteristics and the distribution of risk factors were reported in Table 1: more than 50% of the overall population had arterial hypertension, diabetes and dyslipidemia were present in 10 subjects, 29% of subjects were overweight (BMI ≥ 25 and < 30), 17% obese (BMI ≥ 30) and 31% were smokers. Only 5 (14%) patients had known coronary artery disease.

**Table 1:** Anthropometrical and clinical characteristics of study population

Variables	All subjects (***n*** = 35)
Age (y)	59 (48; 60)
Gender (M)	21 (60%)
BMI (kg/m^2^)	25 (22; 29)
*Risk Factors*	
Smoking habit, n (%)	11 (31%)
Hypertension, n (%)	18 (51%)
Dyslipidemia, n (%)	10 (29%)
Diabetes, n (%)	10 (29%)
Previous cardiovascular disease, n (%)	5 (14%)
*Predisposing factors*	
Gastrointestinal diseases, n (%)	10 (29%)
Autoimmune diseases, n (%)	1 (3%)
Respiratory diseases, n (%)	7 (20%)
Vascular surgery/limb iscaemia, n (%)	1 (3%)
Genitourinary diseases, n (%)	5 (14%)
Orthopedic/skeletal diseases, n (%)	1 (3%)
Haematological diseases, n (%)	10 (29%)
Within 30 days mortality, n (%)	15 (43%)
SBP (mmHg)	120 (108; 134)
DBP (mmHg)	60 (55; 65)
MAP (mmHg)	80 (73; 86)
HR (beats/min)	96 (87; 116)
Epinephrine (ɣ/kg/min)	0.09 (0.04; 0.26)
Norepinephrine (ɣ/kg/min)	0.26 (0.12; 0.49)
TnT (ng/L)	91 (38; 193)
PCT (ng/mL)	34 (9; 100)
NT-ProBNP (ng/L)	12,324 (3135; 29,571)
*Echocardiograghy values*	
VTD (mL)	101 (62; 110)
VTS (mL)	39 (31; 53)
Dtd (mm)	45 (41; 51)
EF (%)	55 (40; 60)

Data are expressed as median and interquartile range (I-III) or as number and percentage. *BMI* body mass index, *SBP* systolic blood pressure, *DBP* diastolic blood pressure, *MAP* mean arterial pressure, *HR* heart rate, *TnT* troponine T, *PCT* pro-calcitonin, *NT-ProBNP* pro b-type natriuretic peptide, *VTD* and *VTS* telediastolic and telesystolic volumes, *EF* ejection fraction

**Fig. 1:**
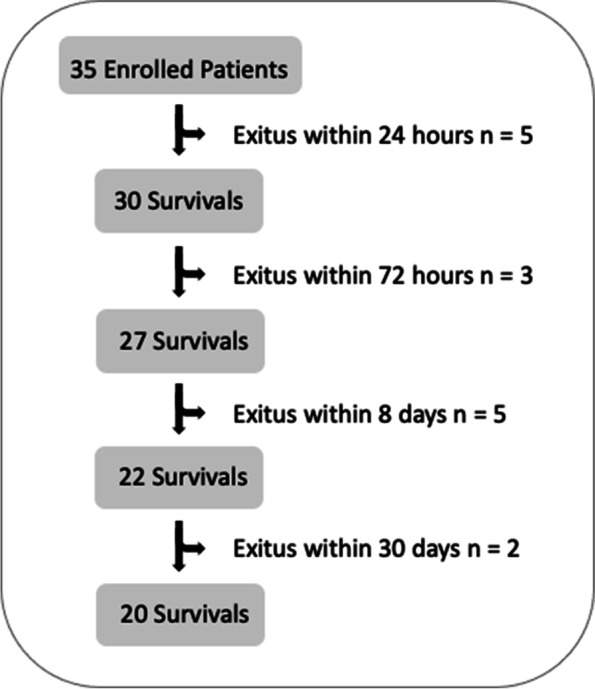
Population and survival

Of the initial sample of 35 patients, 15 (43%) died in the ICU within 30 days as reported in Fig. 1, and other 9 (26%) after a mean of 475 ± 488 days.

### Predictors of in-hospital and 30-day mortality

Table 2 summarizes the main clinical and biochemical variables, comparing patients who died in hospital with those who survived. There was no association between age, gender, oxidative stress markers, arterial stiffness and echocardiographic parameters with mortality in our septic cohort. No association was also found with IL-6 or others inflammatory mediators linked to its pathway. Conversely, MDA, uNEO and Ang-2 appear to be significantly associated to not survived (*P* = 0.074, *P* = 0.099 and *P* = 0.003, respectively). The multivariate Cox’s proportional hazard model, with backward selection method, showed that the only independent prognostic predictor for 30-day mortality was the Ang-2 (HR 1.017 for 500-fold increment, 95% CI 1.000–1.034; *P* = 0.049).

**Table 2:** Univariable Cox regression model

Variables	S group (***n*** = 20)	NS group (***n*** = 15)	P	HR	95%CI
Age, years	56 (46; 68)	64 (54; 73)	0.203	1.027	0.986–1.071
Gender F, n (%)	9 (45%)	5 (33%)	0.563	0.728	0.249–2.131
GSH RB, μmol/L	410 (318; 561)	392 (202; 518)	0.370	0.998	0.995–1.002
GSH TP, μmol/L	4.24 (2.118; 6.228)	2.73 (2.18; 4.18)	0.200	0.833	0.630–1.102
Hcy TP, μmol/L	8.03 (5.92; 14.13)	9.40 (5.48; 15.40)	0.681	1.013	0.952–1.078
CysGly TP, μmol/L	35 (25; 47)	29 (20; 36)	0.454	0.987	0.953–1.022
Cys TP, μmol/L	147 (100; 206)	168 (108; 232)	0.708	1.001	0.998–1.003
MDA, mmol/L	147 (97; 184)	206 (120; 261)	0.074	1.004	1.000–1.008
3-NT nmol/L	12.33 (7.12; 22.65)	12.33 (6.70; 38.10)	0.688	0.999	0.994–1.004
uNEO, μmol/mmol Creat	1.068 (0.528; 1.908)	1.344 (0.822; 3.436)	0.099	1.212	0.964–1.524
IL-6 pg/mL	446 (49; 2540)	1198 (180; 8646)	0.146	1.000	1.000–1.000
sIL-6R pg/mL	31,136 (20,251; 51,166)	33,157 (10,896; 38,651)	0.185	1.000	1.000–1.000
sGP-130 ng/mL	236 (202; 286)	200 (170; 307)	0.685	0.999	0.991–1.006
Ang-1 pg/mL, (500-fold increment)	10,200 (4614; 31,953)	11,062 (4480; 28,900)	0.848	0.999	0.985–1.013
Ang-2 pg/mL, (500-fold increment)	26,950 (10,822; 43,840)	49,048 (33,483; 64,293)	**0.003**	**1.007**	**1.002–1.012**
TnT, ng/L	105 (36; 289)	91 (50; 160)	0.495	1.000	1.000–1.000
NTproBNP, ng/L	19,359 (1991; 31,044)	7558 (3150; 28,820)	0.679	1.000	1.000–1.000
PCT, ng/mL	34 (11; 100)	35 (9; 70)	0.696	0.997	0.983–1.011
PWV, cm/sec	10.3 (8.7; 11.8)	10.8 (8.4; 11.7)	0.931	1.009	0.829–1.228
AX, %	8 (−7; 33)	12 (4; 15)	0.840	1.003	0.974–1.033
VTD, ml	108 (82; 116)	93 (60; 108)	0.220	0.985	0.961–1.009
VTS, ml	39 (32; 49)	39 (29; 61)	0.962	1.001	0.967–1.035
Dtd, mm	49 (41; 54)	45 (40; 49)	0.185	0.926	0.826–1.038
EF, %	55 (40; 64)	55 (39; 61)	0.702	0.992	0.952–1.034
MAPSE, mm	12.5 (12.0; 15.5)	12.5 (11.5; 14.3)	0.779	0.965	0.752–1.239
E, cm/sec	91 (56; 116)	85 (65; 90)	0.466	0.989	0.962–1.018
A, cm/sec	73 (40; 110)	90 (64; 111)	0.472	1.009	0.985–1.032
E/e’	8.55 (5.75; 12.93)	7.40 (5.95; 13.10)	0.998	1.000	0.833–1.201
Left atrial area, cm^2^	21 (17; 26)	19 (17; 23)	0.277	0.920	0.792–1.069
TAPSE, mm	20 (16; 23)	19 (15; 23)	0.958	0.997	0.877–1.132
PAPs, mmHg	36 (35; 46)	34 (30; 41)	0.277	0.941	0.844–1.050

Data are expressed as median and interquartile range (I-III). *GSH* glutathione, *Hcy* homocysteine, *CysGly* cysteinilglycine, *Cys* cysteine, *TP* total plasma, *RB* reduced blood, *MDA* malondialdehyde, 3-*NT* 3-nitrotyrosine, *uNEO* urinary neopterin, *IL-6* interleukin-6, *sIL-6R* soluble IL-6 receptor, *sGP-130* soluble GP-130, *Ang-1* and *-2* angiopoietin-1 and-2, *TnT* Troponin T, *NT-proBNP* pro B-type natriuretic peptide, *PCT* pro-calcitonin, *PWV* pulse wave velocity, *AX* augmentation index, *VTD* and *VTS* telediastolic and telesystolic volumes, *EF* ejection fraction, *MAPSE* mitral anular plane excursion, *TAPSE* tricuspid anular plane excursion, *S* survivors, *NS* non-survivors

**Fig. 2:**
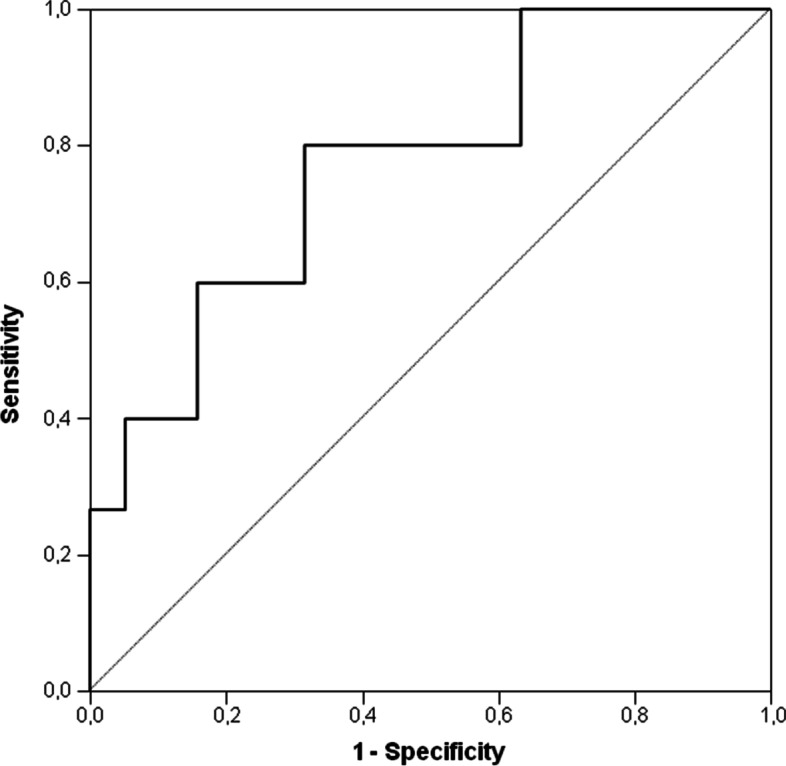
Ang-2 concentrations thresholds for the discrimination between survivors and non survivors at the time of admission in ICU (sensitivity of 80% and a specificity of 68%)

An Ang-2 concentrations ≥ of 33,418 pg/mL was identified as the optimal thresholds for the discrimination between survivors and non-survivors at the time of admission in ICU, with a sensitivity of 80% and a specificity of 68% (Fig. 2). The area under ROC curve was 0.77 with a 95% CI from 0.61 to 0.93 and a *P* < 0.007.

**Fig. 3:**
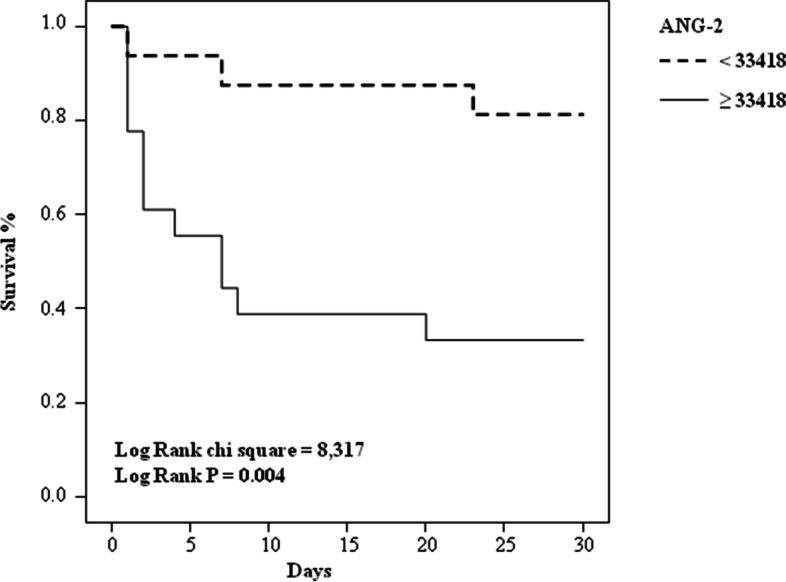
Survival distribution for Ang-2 cut-off

On the basis of Ang-2 cut-off value we performed the Kaplan–Meier analysis. The survival distributions were found to be significantly different (*P* = 0.004) between the 2 groups (Fig. 3): 13 survivors and 3 non-survivors had Ang-2 concentrations < 33,418 pg/mL and 6 survivors and 12 non-survivors ≥33,418 pg/mL.

### Correlation between Ang-2 and other variables

We tested in the overall population the relationship between Ang-2 and other clinical and biochemical variables. Only IL-6 (Rho = 0.471, *P* = 0.005) and uNEO (Rho = 0.423, *P* = 0.025) levels were directed correlated to Ang-2 concentrations. No correlation was found between echo, vascular biomarkers or vasoactive treatments.

## Discussion

The present results confirm that septic shock has a poor in-hospital outcome even when the best of care is implemented. Systolic and diastolic function was comparable between the groups of survivors and non-survivors. When vascular reactivity assessed by pulse wave pressure, no parameter was able to identify patients at risk of death. Moreover, the study showed that Ang-2 is a sensitive biomarker able to predict negative outcome in septic shock patients. No echocardiographic nor vascular parameter were able to predict this outcome.

The angiopoietins are secreted endothelial growth factors that bind to Tie-2 receptors to control endothelial permeability and to regulate angiogenesis [15]. The actions of Ang-1 and Ang-2 oppose each other. Ang-1 keep interendothelial junctions stable and has anti-inflammatory role. Ang-2 (stored in Weibel-Palade bodies of endothelial cells) competes with Ang-1 for binding to Tie-2 increasing vascular leakage by inhibiting autophosphorylation [16]. Increased Ang-2 levels increases permeability and reduces tissue oxygenation and thus leads to hypoxia-induced organ dysfunction [1]. Increased plasma angiopoietin-2 levels are associated with increased fluid overload, hepatic and coagulation dysfunction, acute kidney injury, mortality, and plasma cytokines in human septic shock [1]. Our result is consistent with previous studies, ang-2 permit a more precise risk stratification and was an early prognostic marker of ICU mortality in septic shock [17].

The search for a clinically useful risk stratifier in patients with sepsis and septic shock is still on-going. A measurable parameter able to assess severity of disease and therapeutic efficacy is critical in such a difficult clinical setting with a high mortality rate. Several studies have tried to assess the predictive power of echocardiography and/or circulating biomarkers. In a recent study Landesberg et al. have hypothesized that the assessment of myocardial dysfunction at echocardiography can provide insight into the possible causes of troponin elevation and its association with mortality in sepsis [18]. These Authors conclude that left ventricular diastolic dysfunction and right ventricular dilatation are the echocardiographic variables correlating best with concomitant high-sensitivity troponin-T concentrations. The high concentration of troponin is just a marker of left ventricular diastolic and right ventricular systolic dysfunction and may explain the mortality in severe sepsis and septic shock. The same group had demonstrated, in a cohort of 262 patients, that diastolic dysfunction is common and it is the strongest and independent predictor of early mortality in septic shock, even when corrected with co-morbidities. Moreover, both troponin-T and NT-proBNP are significantly elevated not only in patients with reduced LVEF but also in patients with isolated diastolic dysfunction, when compared with patients having normal systolic and diastolic function [5]. Our results are not consistent with these previous studies. Echocardiographic assessed diastolic and systolic function did not correlate with mortality. The main reason for this discrepancy could be due to the severity of the clinical condition of the sample under investigation. Although significantly smaller than the previous reports, it is a highly selected sample with a severe septic shock. Troponins and NT-proBNP are significantly more elevated in the group of patients who died [19].

The clinical implications of our results may be relevant for the management of septic shock. LV function, both diastolic and systolic, seems to be an organ endpoint and not the cause of death. A novel pattern with several biomarkers is able to identify those patients at higher risk of death. Precision medicine with a tailored approach to risk stratification may change our ability to treat these patients at a very ominous outcome. Echocardiography may not be relevant for the prognosis of patients with septic shock. In most cases LV function is preserved and its assessment does not add critical info for patient risk stratification. PWV parameters are also not associated with mortality in our cohort probably because these variables are influenced by several confounding factors such as pharmacological load, volume filling and artificial ventilation.

Several limitations have to be acknowledged to this study. It is a single center study conducted in a tertiary referral center for sepsis. There is no cost-effectiveness analysis to implement a strategy that may be more expensive than the conventional approach in patients with sepsis. There was no central reading for echocardiography but the same operator analyzed the images and this may have reduced significantly the inter-observer variability. The choice of the biomarker to be used in this setting was arbitrary. There was the need to explore the different factors contributing to the development of organ failure in severe sepsis.

## Conclusions

Septic shock is related to a very ominous prognosis and few tools are available to identify those patients at higher risk of death and to modulate therapy accordingly. Among evaluated biomarkers only Ang-2 was able to identify those patients at risk of death. A more widespread use of biomarkers should be assessed in properly designed trials in order to provide an individual risk stratification to tailor therapy.

## Data Availability

The data presented in this study are available from the corresponding author upon reasonable request.

## Abbreviations

Ang-1 and -2: Angiopoietin-1 and -2
cfPWV: Carotid-femoral pulse wave velocity
CI: Confidence interval
crPWV: Carotid-radial pulse wave velocity
Cys: Cysteine
CysGly: Cysteinylglycine
CRP: C-reactive protein
2D: Two-dimensional
GSH: Glutathione
Hcy: Homocysteine
HPLC: High performance liquid chromatograpy
ICU: Intensive care unit
IL-6: Interleukin-6
LV: Left ventricular
MDA: Malondialdehyde
3-NT: 3-nitrotyrosine
PCT: Pro-calcitonin
RB: Reduced blood
ROC: Receiver operating characteristic
sGP-130: Soluble G-protein-130
sIL-6R: Soluble interleukin-6 receptor
TDI: Tissue doppler imaging
TP: Total plasma
uNEO: Urinary neopterin
